# African network for statistical excellence in health research

**DOI:** 10.7189/jogh.16.03022

**Published:** 2026-07-21

**Authors:** Michal Ordak, Lise Korsten, George Oyamo Osanjo, Adey Desta

**Affiliations:** 1 Centre of Regenerative Medicine, Medical University of Bialystok, Poland; 2 Department of Science, Technology and Innovation, Pretoria, South Africa; 3 African Academy of Sciences, Nairobi, Kenya; 4 Department of Pharmacology, Faculty of Health Sciences, University of Nairobi, Kenya; 5 Department of Microbial Sciences and Genetics, College of Natural and Computational Sciences, Addis Ababa University, Ethiopia

**Keywords:** Africa, biostatistics capacity building, statistical training networks

## Abstract

Despite increasing research output across Africa, weaknesses in statistical methodology continue to compromise the quality, reproducibility, and practical value of health research. Although substantial investments have been made in research capacity strengthening, limitations in statistical training and access to biostatistical expertise remain widespread. Existing initiatives, including regional postgraduate programmes and institution-specific training schemes, have contributed to advances in biostatistical capacity but remain fragmented, geographically limited, and insufficiently integrated into routine research practice. In this viewpoint, we argue that strengthening statistical capacity in Africa now requires a coordinated, continent-wide training network embedded within existing academic and research systems, rather than continued reliance on isolated programmes. The proposed model emphasises scalable training infrastructure, integration of statistical expertise throughout the research process, and structured collaboration between institutions facing similar methodological challenges. Central components include accessible training resources, mentorship, incorporation of statistics into existing curricula, and practical support linked to ongoing research activities. Emphasis is placed on standardising core competencies in statistical practice, including appropriate method selection, assessment of assumptions, transparent reporting, and accurate interpretation of findings. By embedding statistical thinking from study design through publication, the proposed network aims to improve methodological consistency, strengthen research credibility, and enhance the global impact and usability of health research conducted across Africa.

Africa is producing more health research than ever before, but much of it remains methodologically fragile and difficult to reproduce. Despite sustained investment in capacity building, persistent gaps in statistical expertise and training continue to affect research practice across many settings [1]. However, research capacity and statistical infrastructure vary substantially across African countries, reflecting differences in resources, training systems, and institutional development. This diversity suggests that a single unified model must be adaptable to different baseline levels of statistical capacity across settings.

Systematic evaluations of health science research from African settings have also identified limitations in study design and statistical practice, further highlighting the need for strengthened biostatistical capacity and methodological support within research institutions [2]. Evidence from African institutions indicates a continuing demand for practical biostatistics training, statistical consultation services, and coordinated support structures that can be integrated into routine research activities [3]. Simultaneously, the rapid expansion of health research and increasingly complex data sets across sub-Saharan Africa has not always been matched by equivalent growth in local analytical capacity. As a result, many institutions continue to face challenges related to statistical supervision, career development opportunities for biostatisticians, and the effective use of statistical analyses to inform research and policy [4].

Africa does not need more short-term fellowships. It needs a coordinated, continent-wide statistical training network embedded within academic and research institutions. These persistent methodological and capacity challenges point to a need for coordinated, institution-wide responses. Different types of statistical challenges require different forms of training and support, ranging from basic study design to more advanced analytical methods. We believe that a coordinated, continent-wide approach to statistical training offers a scalable and institutionally supported solution to strengthening statistical capacity and improving research quality, and is already being developed through consultations with academic leaders, researchers, and trainees from multiple African medical universities.

## LIMITATIONS OF CURRENT INITIATIVES

For over a decade, efforts to address this problem have been ongoing, yet the same challenges persist. Early initiatives highlighted the need to develop local expertise and build sustainable training infrastructure, particularly in response to growing biomedical research activity and increasing demand for in-country biostatistical expertise [5]. Subsequent discussions emphasised the shortage of trained biostatisticians and proposed collaborative regional approaches, including dedicated centres of excellence and specialised training programmes [6]. More recently, programmes such as the Sub-Saharan African Consortium for Advanced Biostatistics have expanded postgraduate training opportunities, supported the establishment of new biostatistics programmes, and strengthened institutional collaboration across multiple countries, but their reach remains limited relative to the scale of need across the continent [7]. Similarly, initiatives such as the Vanderbilt Nigeria Biostatistics Training Program have strengthened capacity through fellowships, workshops, mentorship, and applied research training, but remain geographically and thematically constrained [8].

Existing capacity-building initiatives, therefore, remain limited in scale and geographic coverage relative to the continent-wide need. They represent important progress, but also illustrate a persistent pattern of valuable efforts that are fragmented, time limited, and insufficiently integrated into everyday research practice. Potential barriers to sustained integration include dependence on project-based funding, limited institutional embedding of statistical support services, shortages of dedicated biostatistical positions, and restricted opportunities for long-term mentorship and career development. In our view, without a coordinated system, these limitations will continue to undermine the quality and reproducibility of research across the continent.

## A COORDINATED NETWORK APPROACH

We argue that the next phase of capacity building must move beyond isolated programmes towards a coordinated system. Network-based approaches have been widely used in research capacity-strengthening initiatives to support collaboration, knowledge sharing, mentorship, and standardisation across institutions, providing a potential framework for more sustainable integration of statistical support into routine research practice. Such a network would provide a structured and scalable framework to strengthen statistical capacity across institutions throughout Africa. Its core components would include the development of accessible training resources tailored to the needs of medical researchers, alongside the integration of statistical training into existing academic programmes. Accessible training resources could include recorded lectures, case-based learning modules, practical statistical exercises, and guidance documents that can be incorporated into existing postgraduate and research training programmes. Training content should therefore be structured to address a range of methodological needs, from foundational statistical concepts to more advanced modelling approaches. Crucially, this means embedding statistical expertise early in the research process, not applying it retrospectively. Without such integration, limitations in statistical practice will continue to affect the reliability and usability of research findings.

## INTEGRATION WITH RESEARCH PRACTICE

A key feature of this model is the direct linkage between training and real research activity. Recorded materials and other resources do not function as standalone educational elements, but support the application of statistical principles in ongoing projects, from study design to data analysis and manuscript preparation. In this model, biostatistics becomes a practical research tool, rather than a theoretical requirement. The network would also facilitate collaboration between institutions, which could include regular online methodological workshops, joint review of study protocols and statistical analyses, and cross-institutional mentorship involving both researchers and biostatisticians. Researchers working in similar contexts often encounter comparable methodological challenges. Creating opportunities for shared discussion and structured review of analyses and manuscripts would enable solutions developed in one setting to be adapted in others, improving methodological consistency across institutions.

## STANDARDISING CORE COMPETENCIES

Equally important is the need to standardise core competencies in statistical practice. Rather than focusing on advanced or highly specialised methods, the priority should be the consistent application of fundamental principles. Ensuring that researchers can select appropriate statistical methods, assess underlying assumptions, interpret results correctly, and report analyses transparently would address many of the recurring issues observed in submitted manuscripts. Evidence from biostatistics education shows that approaches based on real research examples improve understanding and confidence compared with purely theoretical teaching [9]. Establishing shared expectations in these areas would also help align the practices of researchers, institutions, and journals.

## MOVING FORWARD

Implementation should be gradual and grounded in practice. Initial pilot activities could focus on integrating statistical support into ongoing research projects, while using the developed training resources in real research settings. Pilot implementation could involve selected academic and research institutions, with evaluation based on participation, utilisation of training resources, engagement in mentorship activities, and integration of statistical support into ongoing research projects. Implementation should therefore be context-sensitive, with adaptation of training intensity and support mechanisms according to local institutional capacity and resources. It would follow a phased approach, beginning with pilot institutions and expanding iteratively based on evaluation outcomes over a defined multi-year period. This would allow the model to evolve iteratively and scale across institutions while maintaining methodological consistency.

Future development of the network will also require consideration of governance structures, sustainable funding mechanisms, quality assurance processes, multilingual delivery of training resources, and differences in institutional capacity across participating institutions. The network should also be designed to accommodate diverse institutional contexts across the continent. This may include the use of downloadable and low-bandwidth training resources, multilingual delivery where feasible, and mechanisms to support participation from under-resourced institutions to minimise geographical and institutional inequities in access to training and statistical support.

The success of the model will depend on sustained institutional engagement, availability of statistical expertise, and adequate resource support – factors which should be considered during future implementation and evaluation. The network could be coordinated through a consortium of participating academic and research institutions. Funding would likely be based on a mixed model combining institutional contributions, competitive research and capacity-building grants, and support from international partners. The consortium would provide overall governance, including coordination of training standards, oversight of activities, and strategic alignment across participating institutions. Core activities would include the development of shared training resources, mentorship, statistical support, and collaborative methodological review. These activities would support the development of statistical competencies and strengthen the integration of statistical expertise into ongoing research.

Quality assurance would be ensured through standardised training materials, periodic review of content, and agreed reporting and accountability mechanisms across participating institutions. Expected outcomes would include improved methodological practice, more consistent application of statistical principles, and enhanced quality and reproducibility of health research across participating institutions. Quality could be supported through shared standards and periodic review of training materials and activities. We suggest that the next step is to prioritise coordinated implementation across institutions, supported by academic leadership and embedded within existing research structures. The question is no longer whether statistical capacity in Africa needs strengthening, but how to achieve this in a coordinated, scalable, and embedded way. A network-based approach offers a clear and practical path forward. Continuing to rely on fragmented, short-term initiatives will only perpetuate the limitations they are intended to solve. Moving from isolated programmes to an integrated system is essential to improve the credibility, usability, and global impact of health research conducted across the continent.

## Data Availability

**Data availability:** No new data were generated during the preparation of this viewpoint.
